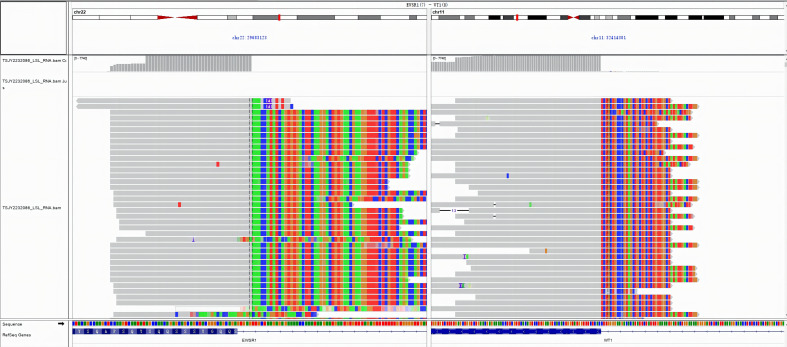# Abdominopelvic desmoplastic small round cell tumor with metastasis: A case report and literature review

**DOI:** 10.1097/MD.0000000000037664

**Published:** 2024-04-05

**Authors:** Guoyong Chen, Qian Zhang, Dong Xia

**Affiliations:** a Department of Clinical Medicine, Southwest Medical University, Luzhou, Sichuan Province, China; b Gastrointestinal Group, Department of General Surgery, Affiliated Hospital of Southwest Medical University, Luzhou, Sichuan Province, China.

**Keywords:** abdominopelvic cavity, case report, desmoplastic small round cell tumor, soft tissue sarcoma

## Abstract

**Rationale:**

Desmoplastic small round cell tumor (DSRCT) is a rare and rapidly metastasizing soft tissue sarcoma, distinguished by its unique cell morphology and pleomorphic differentiation.

**Patient concerns:**

This report describes the case of an 18-year-old male diagnosed with abdominopelvic DSRCT exhibiting metastases to the peritoneum, liver, pleura, bone, and muscle. The patient primarily presented with symptoms of incomplete intestinal obstruction and an abdominal mass.

**Diagnoses:**

Colonoscopy revealed lumen stenosis caused by external compression mass. Contrast-enhanced computed tomography and ^18^F-fluorodeoxyglucose positron emission tomography/computed tomography revealed multiple lesions in the abdominopelvic cavity. A needle biopsy of an abdominal wall lesion established it as a malignant tumor, origin unknown. Immunohistochemical staining post-surgery showed positive results for Cytokeratin (CK), CK7, Desmin, Vimentin, Caudal type homeobox 2 (CDX2), and Ki-67. Fluorescence in situ hybridization analysis revealed an Ewing sarcoma breakpoint region 1/EWS RNA binding protein 1 (EWSR1) rearrangement, and next-generation sequencing identified an EWSR1-Wilms tumor protein 1 (WT1) gene fusion.

**Interventions:**

The patient underwent laparoscopic exploratory surgery, which encompassed biopsy, ascites drainage, adhesion lysis, reinforcement of weakened sections of the small intestinal walls, and repositioning of twisted intestines. Postoperatively, the treatment protocol included fasting, rehydration, gastrointestinal decompression, and parenteral nutrition. However, the patient did not received chemotherapy.

**Outcomes:**

The patient declined further treatment and deceased in early November.

**Lessons:**

This case highlights the nonspecific nature of DSRCT symptoms. In clinical practice, it is crucial to meticulously evaluate unexplained intestinal obstruction in young patients, considering DSRCT as a differential diagnosis to avoid delays in diagnosis.

## 1. Introduction

Desmoplastic small round cell tumor (DSRCT) is a highly malignant and rare tumor, predominantly found in the abdominal and pelvic regions.^[1]^ It primarily affects adolescents and children, with patient ages ranging from 3 to 52 years and an average age of 22. DSRCT represents <1% of all soft tissue sarcomas. Initially described by Gerald and Rosai in 1989, it was officially named in 1991.^[2]^ Characteristically, DSRCT comprises small round cells of varying sizes within a densely proliferative fibrous stromal matrix.^[3]^ The cells display polyphenotypic differentiation, expressing markers like cytokeratins AE1/AE3, epithelial membrane antigen, desmin, neuroendocrine markers (CD57, neuron-specific enolase), and Wilms’ tumor protein 1 (WT1).^[4]^ In cytogenetics, its hallmark is the Ewing sarcoma breakpoint region 1/EWS RNA binding protein 1 (EWSR1)-WT1 fusion gene, due to a translocation between chromosomes 11 and 22 (t(11;22)(p13:q12)).^[5]^ Patients typically present with abdominal discomfort, celialgia, and palpable masses, but bowel obstruction symptoms are uncommon, often resulting in delayed diagnosis due to their nonspecific nature.^[6]^ This report discusses a case of metastatic DSRCT in the abdominopelvic cavity, associated with incomplete intestinal obstruction.

## 2. Case report

An 18-year-old male presented with symptoms of upper abdominal pain, abdominal distension, difficulty in defecation, and a palpable abdominal mass persisting for nearly 6 months. The patient underwent an abdominal CT scan at another hospital, which revealed an abdominal mass of unknown diagnosis, leading to a transfer to our institution. His medical history included a spontaneous pneumothorax 2 years prior and a right knee dislocation due to physical trauma 1 year ago, both conservatively treated. He had no exposure to toxic or radioactive substances, and his family history was unremarkable. Physical examination showed normal vital signs, with audible bowel sounds. The abdomen appeared flat with a dough kneading sensation, scattered tenderness, but no muscular spasm or rebound tenderness. A firm, irregular, mobile mass, approximately 5 cm × 4 cm, was palpable in the epigastric region and non-tender. Additionally, 4-5 grape-like clusters of nodules, firm and smooth, were detected in the umbilical and hypogastric regions, with no tenderness. Mild percussion pain was noted in the liver area. Digital rectal examination identified a suspicious mass in the pelvis, causing external compression.

**Figure 1.**
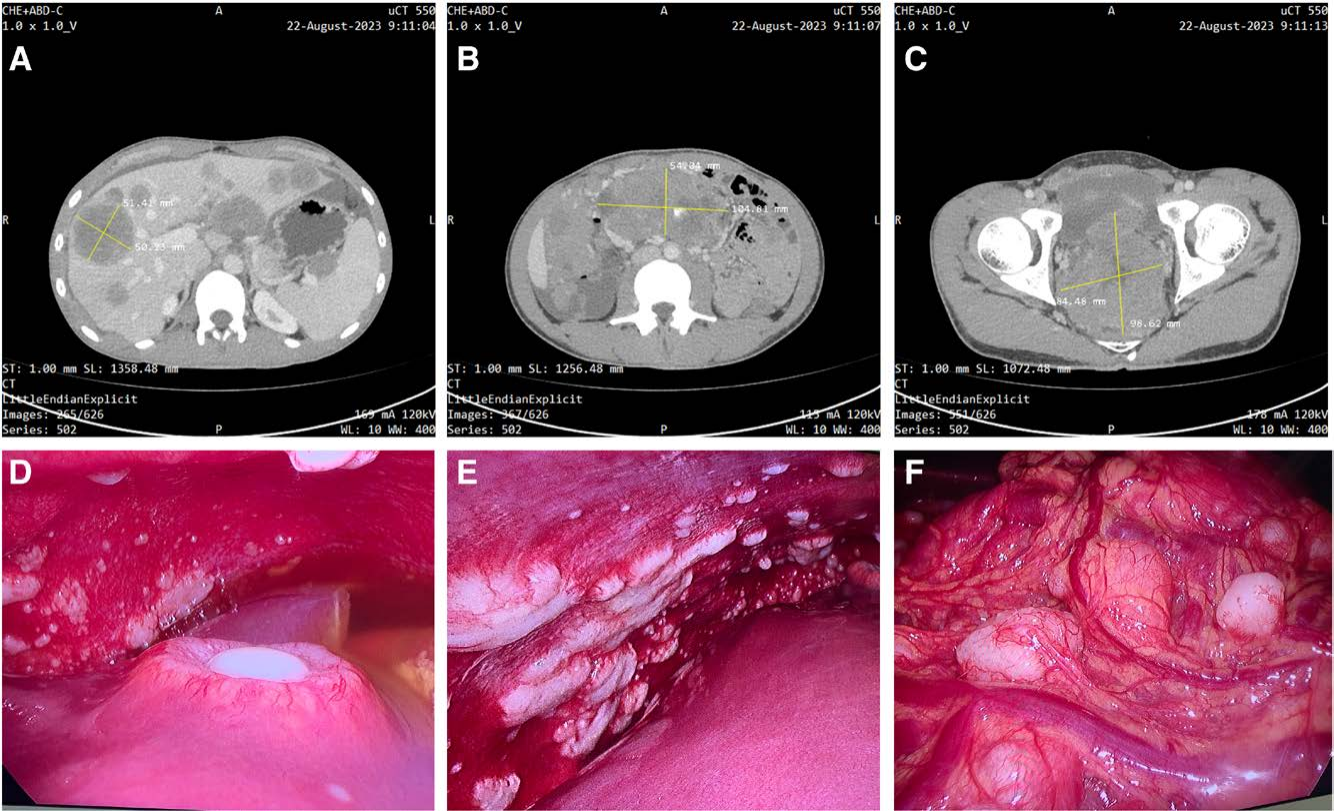
A selection of ceCT images and intraoperative observations. The ceCT revealed tumors in the abdominal and pelvic regions measuring approximately 104.81 mm × 54.04 mm and 98.62 mm × 84.48 mm, respectively. These masses displayed heterogeneous density and non-uniform enhancement. Additionally, multiple hepatic lesions with inhomogeneous density and lower enhancement were noted, the largest being about 51.41 mm × 50.23 mm in cross-section (A − C). The liver surface was irregular, featuring multiple reddish, volcano-like tumors. Additionally, numerous firm, gray-white nodules of irregular sizes, resembling grape clusters, were observed on the greater omentum and abdominal wall (D − F). ceCT = contrast-enhanced computed tomography.

Initial laboratory tests revealed elevated neutrophil ratio (NEU-R), lactate dehydrogenase, and carbohydrate antigen 125 (CA125), accompanied by a decreased lymphocyte ratio (LYM-R). All other test indices were within normal limits (Table 1). CT enhanced scan revealed multiple abdominopelvic masses, the largest in upper-middle abdomen measuring approximately 10.6 × 5.7 cm with mixed density. Secondary sites were observed in the liver, pancreas, gastrosplenic recess, left perihepatic space, diaphragm, right pleura, and the sixth right rib (Fig. 1A − B). Detailed findings from the subsequent PET/CT examination are presented in Figure S1, http://links.lww.com/MD/M18. Colonoscopy disclosed a protruding lesion situated about 6 cm to 15 cm from the anus causing intestinal stenosis. Needle biopsy of the abdominal wall lesion confirmed its malignancy, with the primary site undetermined. Laparoscopic exploration identified 4 moderate-sized, relatively isolated omental metastatic nodules for biopsy. The surgery also entailed adhesion lysis, reinforcing weakened areas of the small intestinal walls, freeing twisted intestine, and placing an abdominal drain. Surgical findings included approximately 500 mL of ascites and an irregular liver surface with multiple volcano-like lesions. Numerous gray-white nodules, varying in size and akin to grape clusters, were distributed across the anterior wall of the stomach, gallbladder, omentum, small intestine, colon, and abdominal pelvic wall (Fig. 1D − F). The surgical duration was approximately 220 minutes. Amount of bleeding was about 5 mL. Postoperatively, the patient underwent a treatment regimen comprising fasting, rehydration, gastrointestinal decompression, and parenteral nutrition. Enteral nutrition was gradually resumed on the third day following surgery without further obstruction symptoms. However, the patient self-discharged on the seventh postoperative day and did not receive subsequent chemotherapy, ultimately succumbing to their condition in early November.

**Table 1:** Partial laboratory test results.

Items	Results	Units	Reference range
Routine blood test			
WBC	9.72	10^9^/L	4.1–11.0
NEU	7.50	10^9^/L	1.8–8.3
NEU-R	77.2%↑	%	37–77
LYM	1.32	10^9^/L	1.2–3.8
LYM-R	13.6%↓	%	17–54
HGB	151	g/L	129–172
PLT	410	10^9^/L	150–407
Biochemistry test			
ALT	22.2	U/L	7–43
AST	25.3	U/L	12–37
TBIL	15.8	µmol/L	0–23
LDH	307.3↑	U/L	120–250
GGT	28.8	U/L	8–40
ALP	124.5	U/L	51–202
Urea	3.82	mmol/L	2.7–7.0
Crea	59.8	µmol/L	52–101
GFR	142.3	mL/min	75–145
Serum tumor markers			
AFP	1.94	ng/mL	0.00–10.00
CEA	0.58	ng/mL	0.00–6.00
FER	274.75	ng/mL	25.00–280.00
CA199	3.81	U/mL	0.00–37.00
CA242	0.99	U/mL	0.21–25.00
CA724	1.05	IU/mL	0.00–6.00
PIVKA-II	6.90	Mau/mL	0.00–37.80
CA125	454.53↑	U/mL	0.00–35.00
PSA	0.228	ng/mL	0.00–3.09

AFP = alpha-fetoprotein, ALP = alkaline phosphatase, ALT = alanine aminotransferase, AST = aspartate aminotransferase, CA = carbohydrate antigen, CEA = carcinoembryonic antigen, FER = ferritin, GFR = glomerularfiltrationrate, GGT = gamma glutamyl transferase, HGB = hemoglobin, LDH = lactate dehydrogenase, LYM = lymphocyte, NEU = neutrophil, PIVKA-II = protein Induced by Vitamin K Absence or Antagonist-II, PLT = platelet, PSA = prostate specific antigen, TBIL = total bilirubin, WBC = white blood cell.

**Figure 2.**
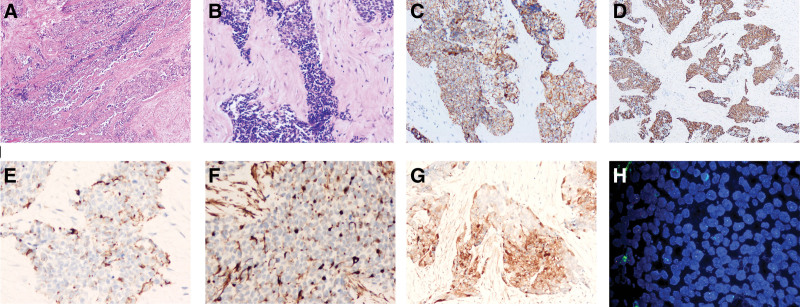
Images of immunohistochemistry staining and fluorescence in situ hybridization. (A) HE × 100, (B) HE × 100, (C) CK (+), (D) CK7 (+), (E) Desmin (+), (F) Vimentin (+), (G) NSE (partially+). The fluorescence in situ hybridization revealed a positive EWSR1 rearrangement. CK = cytokeratin, EWSR1 = Ewing sarcoma breakpoint region 1/EWS RNA binding protein 1, HE = hematoxylin-eosin staining, NSE = neuron-specific enolase.

The postoperative immunohistochemistry (IHC) results indicated positivity for Cytokeratin (CK) and CK7, while CK20, villin, special AT-rich sequence-binding protein 2, leucocyte common antigen, chromogranin A, Synaptophysin, cluster of differentiation 56 (CD56), podoplanin, CK5/6, calretinin, and WT1 were negative. CDX2 and Ki-67 also tested positive, with Ki-67 exhibiting a 40% expression rate. Desmin and Vimentin showed positive membranous patterns, and neuron-specific enolase was partially positive (Fig. 2A − G). The Vysis EWSR1 Break Apart fluorescence in situ hybridization Probe Kit, targeting the EWSR1 gene at the 22q12 chromosomal site, indicated a positive EWSR1 rearrangement in 90% of the cells (Fig. 2H). Frozen specimens were analyzed for fusion gene sequencing, which confirmed the presence of an EWSR1-WT1 fusion, further corroborating the aforementioned diagnosis (Fig. S2, http://links.lww.com/MD/M19).

## 3. Discussion

DSRCT most commonly occurs in adolescents and children, and is rare in the elderly.^[7]^ The incidence of DSRCT shows no significant racial variation but has a male predominance, with a male-to-female ratio of approximately 3 to 5:1. The origin of DSRCT is still unclear, though it is thought to possibly stem from primitive progenitor cells capable of pleomorphic differentiation.^[8]^ Tumor cells in DSRCT exhibit characteristics of epithelial, muscular, stromal, and neural differentiation. The tumor microenvironment is marked by an increase in fibrous connective tissue, occasionally accompanied by hyaline and mucoid degeneration.^[9]^ Cytogenetically, DSRCT is characterized by the chromosomal translocation t(11;22)(p13;q12), which results in EWSR1-WT1 fusion gene. Diagnosis of DSRCT is reliant on histopathology, IHC, fluorescence in situ hybridization, and karyotype analysis.^[10]^ The differential diagnosis for primary intra-abdominal DSRCT encompasses neuroblastoma, gastrointestinal stromal tumor (GIST), Ewing sarcoma, mesenteric lymphoma, and malignant mesothelioma. Currently, DSRCT does not have a standardized staging system. Clinically, the Peritoneal Cancer Index scoring system and the MD Anderson Cancer Center staging system are frequently used, though the latter validation is pending.^[6]^ R0 resection is a curative approach for localized lesions in DSRCT. However, the tumor aggressive nature often results in extensive abdominopelvic infiltration at diagnosis, limiting most patients to limited cytoreductive surgery or procedures addressing complications like intestinal perforation and obstruction. DSRCT shows dose-dependent sensitivity to alkylating agents and other cytotoxic drugs. The standard treatment regimen includes VAI (vincristine, doxorubicin, and ifosfamide). Current clinical trials are exploring experimental treatments such as the receptor tyrosine kinase (RTK) inhibitor Pazopanib, dopamine-like receptor 2 antagonist ONC201, intraperitoneal radioimmunotherapy with 131I-omburtamab, and hyperthermic intraperitoneal chemotherapy.^[11–14]^ DSRCT predominantly affects young males post-puberty, with androgens playing a role in its progression.^[15]^ Salah-Eddine et al employed enzalutamide and androgen receptor-directed antisense oligonucleotides (AR-ASO) to inhibit DSRCT cell growth induced by 5α-dihydrotestosterone, significantly decreasing xenograft tumor burden and highlighting the efficacy of androgen-targeted therapies.^[16]^ The low mutation burden of DSRCT, contributing to its immunologically “cold” status, indicates that transforming it into a “hot” status might be an effective treatment approach.^[17]^ Unfortunately, DSRCT prognosis remains bleak, with a 5-year survival rate below 15%, and most patients die within 1 to 2 years post-diagnosis.^[18]^

## 4. Conclusions

In our case, the patient presented with an advanced-stage tumor exhibiting multisite and organ invasion. Due to the nonspecific clinical and radiological features of DSRCT, accurate diagnosis necessitates pathology, IHC, and karyotype analysis. Consequently, despite its rarity, DSRCT should be considered in the differential diagnosis of metastatic tumors.

## Acknowledgments

We extend our sincere gratitude to all individuals who contributed to this article.

## Author contributions

**Conceptualization:** Dong Xia.

**Data curation:** Guoyong Chen, Qian Zhang.

**Formal analysis:** Guoyong Chen.

**Writing – original draft:** Guoyong Chen.

**Writing – review & editing:** Qian Zhang, Dong Xia.

## Supplementary Material

**Figure:**
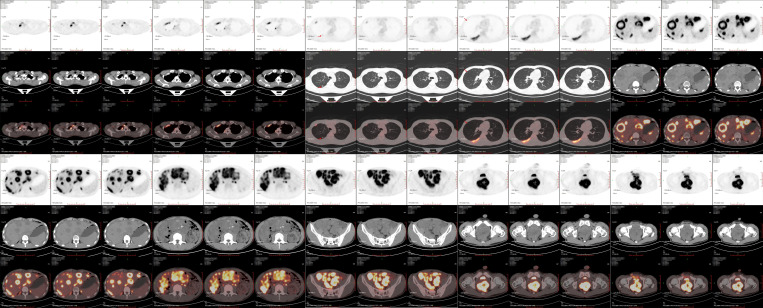


**Figure:**